# Pixel Color Clustering of Multi-Temporally Acquired Digital Photographs of a Rice Canopy by Luminosity-Normalization and Pseudo-Red-Green-Blue Color Imaging

**DOI:** 10.1155/2014/450374

**Published:** 2014-09-15

**Authors:** Ryoichi Doi, Chusnul Arif, Budi Indra Setiawan, Masaru Mizoguchi

**Affiliations:** ^1^Graduate School of Agricultural and Life Sciences, The University of Tokyo, 1-1-1 Yayoi, Bunkyo-ku, Tokyo 113-8657, Japan; ^2^Department of Civil and Environmental Engineering, Bogor Agricultural University, Kampus IPB Darmaga, Bogor 16680, Indonesia

## Abstract

Red-green-blue (RGB) channels of RGB digital photographs were loaded with luminosity-adjusted R, G, and completely white grayscale images, respectively (RGwhtB method), or R, G, and R + G (RGB yellow) grayscale images, respectively (RGrgbyB method), to adjust the brightness of the entire area of multi-temporally acquired color digital photographs of a rice canopy. From the RGwhtB or RGrgbyB pseudocolor image, cyan, magenta, CMYK yellow, black, L*, a*, and b* grayscale images were prepared. Using these grayscale images and R, G, and RGB yellow grayscale images, the luminosity-adjusted pixels of the canopy photographs were statistically clustered. With the RGrgbyB and the RGwhtB methods, seven and five major color clusters were given, respectively. The RGrgbyB method showed clear differences among three rice growth stages, and the vegetative stage was further divided into two substages. The RGwhtB method could not clearly discriminate between the second vegetative and midseason stages. The relative advantages of the RGrgbyB method were attributed to the R, G, B, magenta, yellow, L*, and a* grayscale images that contained richer information to show the colorimetrical differences among objects than those of the RGwhtB method. The comparison of rice canopy colors at different time points was enabled by the pseudocolor imaging method.

## 1. Introduction

Observation of plant color enables suitable plant management measures [1]. Spatial and temporal changes in colors of leaf, flower, fruit, and other plant organs are significantly related to plant nutrition [2], success/failure in plant protection [3], weather stress [4], occurrence of invasive weed species [5], and other types of abnormalities. Various digital observation tools are commonly available and can assist in plant management by providing color digital photographs of plants. The quantification of changes in plant color is difficult, however, because the brightness of the imaging targets varies with photograph acquisition time [6]. Furthermore, there is no correlation among the (intensity) axes of the color components, red-green-blue (RGB), and others [7]. Thus, adjusting the brightness of the entire area of a single digital photograph is difficult [8].

A method to avoid this difficulty was recently demonstrated [9]. An RGB digital photograph consists of data on grayscale intensity between 0 (complete black) and 255 (complete white) for R, G, and B channels [10]. The R, G, and B channels are loaded with R, G, and B grayscale images, respectively. Adjusting the intensity values of pixels of R and G grayscale images of an RGB digital photograph provides the brightness-adjusted R and G grayscale images [11]. The adjustment bases on the fact that the intensity values of R and G have strong correlations to brightness and are thus easy to adjust. However, B has poor correlations to brightness and the intensity of R and G. No other components of the other color models are as precisely brightness adjustable as R and G [7]. To replace the B grayscale image with a white mat (RGwhtB pseudo-RGB color method) may overcome this challenge. Many field and natural plant organs are greenish [2], reddish [12], yellowish [13], or something in between [14]. Yellowness is determined by redness and greenness [15]. The intensity of R and G describes a large portion of information on the colors of plant organs. Thus, the quantification of the intensity values of these visible colors (R and G) should aid significantly in the color observation of plants [16] although invisible (near)-infrared spectra can also provide significant information.

In this context, the first objective of this study was to examine the RGwhtB method in the quantitative color profiling of multi-temporally acquired digital photographs of a rice canopy. Digital photographs of a rice canopy in a paddy field were acquired within a single rice crop season from late August to early December 2011 in Indonesia. Pixels in the acquired digital photographs of the rice canopy were clustered based on their color profiles provided by the method. Another pseudo-RGB color method was developed by replacing B grayscale images with RGB yellow grayscale images by merging brightness-adjusted R and G grayscale images at the same weights [15] (RGrgbyB pseudo-RGB color method). The RGrgbyB method was also used in color profiling of the rice canopy. The second objective was to determine rice growth stages based on the clustering results. The pseudo-RGB methods resulted in different clustering patterns. The pseudo-RGB methods were compared in terms of performance. The differences between the pseudo-RGB methods are discussed herein. 

## 2. Materials and Methods

### 2.1. Site Description

**Figure 1:**
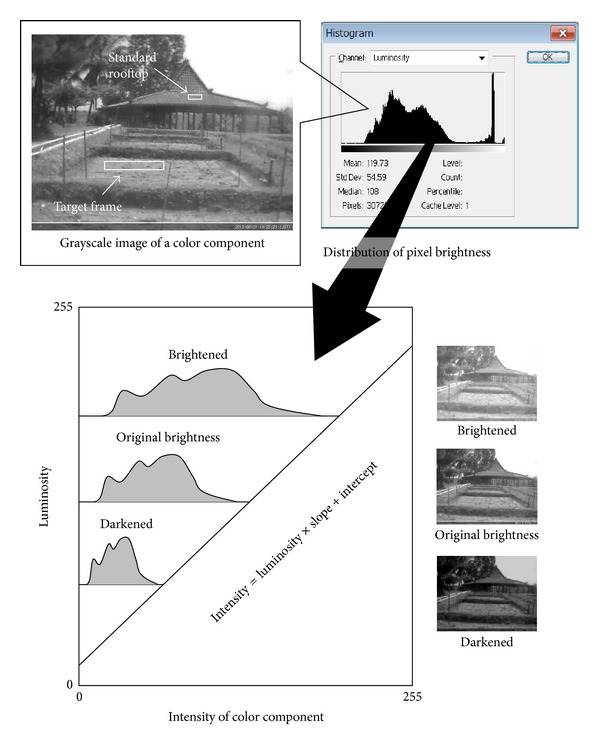
Luminosity-adjustment of grayscale intensity for comparison of multi-temporally acquired digital photographs.

Details of the study site are described elsewhere [17]. The paddy field was located in the Nagrak District of Sukabumi, Indonesia (6°50′42.5′′S, 106°48′20.2′′E). In this study, a rooftop was used as a standard (Figure 1). The digital photographs, as shown in Figure 1, enabled visual confirmation of the aforementioned rooftop and paddy field. The soil texture was silty clay. On 20 August 2011, the field was planted with the local variety of rice (*Oryza sativa *L.), Sintanur. We used the following practices: single planting of young seedlings (10 days after sowing), spaced at 30 cm × 30 cm, applying an organic fertilizer at 7 ton/ha, but no chemical fertilizer. The paddy field was watered according to a non-flooded irrigation system [18, 19]. The silty clay soil was kept moist but with no standing water. The rice was harvested on 12 December 2011. 

### 2.2. Digital Photography and Handling of Digital Photographs

In this study, digital photographs of the paddy field were used. The photographs were captured using a surveillance camera (UCAM-DLO130, Elecom, Osaka, Japan). Photographs were captured daily between 14.00 and 14.30 and stored on a hard disk between 21 August 2011 and 15 December 2011. The rooftop and the paddy field were located within a single scene (Figure 1). When the photograph was captured, data regarding the values of the red-green-blue (RGB) color intensity were generated. The image datum was then pasted into a new file window of Adobe Photoshop 7.0. In another layer overlapping the paddy photograph, frames were set on the rooftop and the rice canopy (Figure 1). The number of spectral-profiled pixels was 168 for the rooftop as the standard and 2,516 for the rice canopy in the target canopy frame. For the pixels in the rooftop frame, the intensity values of R and G were read [20]. For R or G, a value between 0 (darkest) and 255 (saturated and most colorful) was reported. In this study, the AdobeRGB color space was chosen as one of the RGB color spaces. As a measure of brightness, luminosity was also read for the pixels of the rooftop and was averaged.

Using the statistical software SPSS 10.0.1 (SPSS Inc.), linear regression analysis of the luminosity and intensity of the RGB color component was performed to examine if the intensity values of R, G, and RGB yellow could be luminosity-normalized.

Grayscale images that show the intensity values of R and G were prepared [21]. Each grayscale image was subjected to brightness adjustment of the entire image [11]. Luminosity 120-normalization [14] was applied as the method for normalization of brightness of the entire area of the grayscale image. Next, the grayscale image of the intensity values of RGB yellow for the pixels was prepared by merging the luminosity 120-normalized R and G grayscale images at the same weights as described in the manufacturer's instructions [10].

A red-green-white B (RGwhtB) image was prepared by using the luminosity 120-normalized R and G grayscale images and a white mat instead of the B grayscale image. Likewise, a red-green-RGB yellow (RGrgbyB) image was prepared by using the luminosity 120-normalized R, G, and RGB yellow grayscale images.

The authors prepared an RGwhtB or an RGrgbyB image file in which the luminosity 120-normalized pixels of the target frame and the rooftop were chronologically pasted to show temporal changes in the color profile of the rice canopy in the luminosity 120-normalized images. In the same image, the Microsoft Office gamut was pasted to monitor how the pixels of various colors are shown by the handling processes. From the RGwhtB or the RGrgbyB JPEG image, the grayscale images that show the intensity values of B, cyan (C), magenta (M), yellow (Y), key black (K), and lightness (L*) and the values of a* and b* were prepared [21]. CMYK images were generated with the International Color Consortium profile of US Web Coated (SWOP) v2 for digital output such as color printing.

### 2.3. Data Analyses

The R, G, B, and other grayscale images were used in the clustering of the pixels by running the pixel analysis software, MultiSpec 3.3 (Purdue Research Foundation). The iterative self-organizing data analysis technique [22] was applied to cluster the pixels in the JPEG image into color clusters. The minimum cluster size was 12 pixels, the first critical distance was 33 Euclidean distance, and the other critical distances were 66 Euclidean distances.

Images of the target canopy frame were cluster-profiled based on the distribution of color clusters. The Shannon diversity index for the canopy [23] was determined for the target canopy using the following equation:

(1) $$\begin{array}{c} \text{Shannon}\;\text{diversity}=-\Sigma pi\ln pi, \end{array}$$

where *pi* is the proportional abundance for the *i*th cluster over the total pixels for the canopy. Relative proximity among clustering patterns for the time points was quantified by performing multidimensional scaling using the statistical software SPSS 10.0.1.

## 3. Results and Discussion

Forty-nine photographs were obtained within the period. Mean luminosity values for the rooftop within the period ranged between 100 and 135. For the rooftop, the following linear relationships were obtained between luminosity and the intensity of R, G, and RGB yellow.

 Intensity of R = 1.09× luminosity − 2.44 (*R* ^2^ = 0.835, *P* < 0.001).
 Intensity of G = 0.988× luminosity − 4.41 (*R* ^2^ = 0.948, *P* < 0.001).
 Intensity of RGB yellow = 2.08 × luminosity − 6.85 (*R* ^2^ = 0.980, *P* < 0.001).

**Figure 2:**
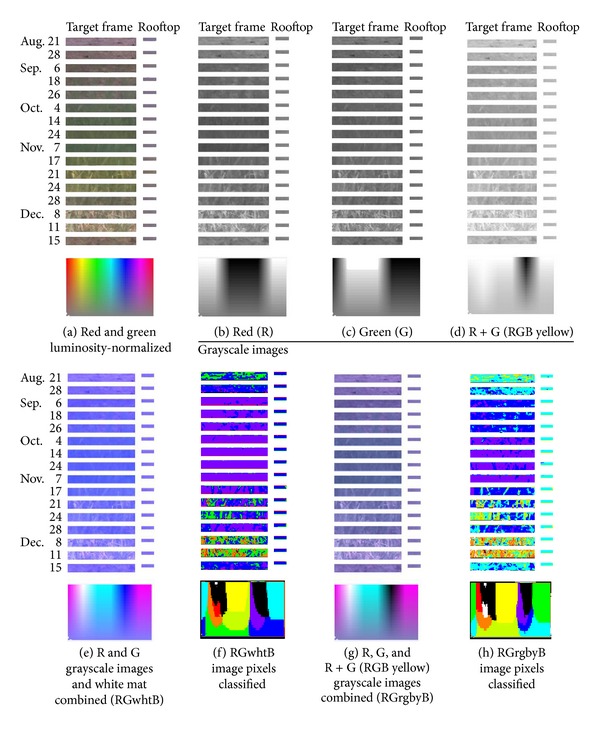
JPEG images for observation of changes in rice canopy color indicated as pixels of multi-temporally acquired digital photographs (a) and the derivative JPEG images.

Using the above equations, in each RGB digital photograph, the R and G grayscale images were luminosity 120-normalized, as shown in Figure 1.  Figure 2(a) was obtained by copying pixels in the target canopy frame and the standard rooftop in the R- and G-luminosity 120-normalized RGB photographs on the selected dates and pasting them in a single image. In the luminosity 120-normalized RGB photograph, the mean intensity values of R and G for the rooftop were 128 and 114, respectively. Then, from Figure 2(a), in which 16 pairs of target frames and rooftops were pasted, luminosity 120-normalized R and G grayscale images were obtained (Figures 2(b) and 2(c)). A pseudo-RGB image was prepared by substituting the B grayscale image of Figure 2(a) with a white mat (RGwhtB, Figure 2(e)) or with the RGB yellow grayscale image (RGrgbyB, Figure 2(g)).

In the RGB image (Figure 2(a)), changes in color were visible for the rooftop, despite the luminosity 120-normalization of the R and G grayscale images. This indicates that the intensity of B changed so significantly that the changes became visible to the human eye. Changes in the grayscale intensity of R, G, and RGB yellow for the rooftop, however, were difficult to perceive (Figures 2(b), 2(c), and 2(d)). The grayscale images of R, G, and RGB yellow also indicate significant changes in the color profile of the pixels in the target canopy frame. On 8 and 11 December, the entire target canopy eventually became light-colored according to the R, G, and RGB yellow grayscale images (Figures 2(b), 2(c), and 2(d)) due to late leaf senescence resulting from the rice farming practices [24].

**Figure 3:**
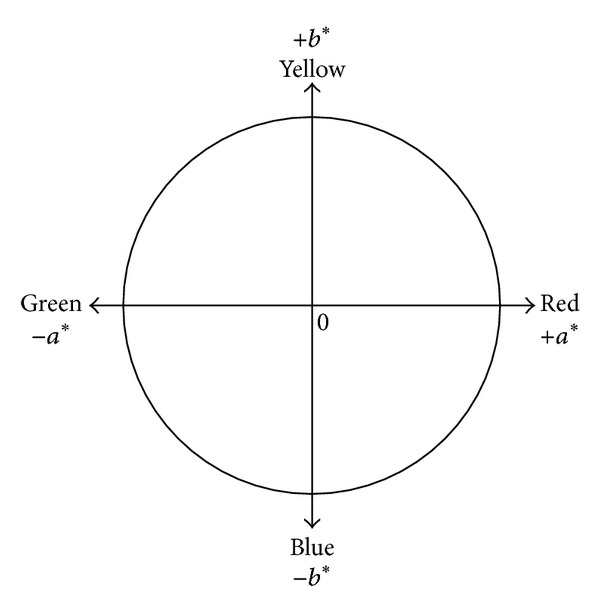
Axes of a*and b*of the L*a*b*color model as measures of redness/greenness and yellowness/blueness, respectively.

By clustering pixels in Figures 2(e) and 2(g), the mean (intensity) values of R, G, B, and the other color components were determined for each color cluster (Figures 2(f) and 2(h), Table 1). Values of a* and b* are shown as those between green (0) and red (255) and blue (0) and yellow (255), respectively (Figure 3, [25]). By adopting a criterion to select the most common pixel color clusters (100 pixels or more in the 16 target frames), five (RGwhtB) and seven (RGrgbyB) color clusters were selected to describe changes in color of the target canopy within the period of the observation (Table 1). The other pixels belonging to the nonselected minor clusters will be referred to as “the other pixels” hereafter. Values of the maximum − the minimum (ranges) of the (intensity) values of the color components tended to be larger for the RGrgbyB method than the RGwhtB method. These differences between the methods suggest that the RGrgbyB method was more informative in indicating temporal changes in pixel color in the target frame than the RGwhtB method.

Differences between canopy clustering patterns for September and October were clearly indicated in the color cluster image for the RGrgbyB method (Figure 2(h)). The color cluster image given by the RGwhtB method, however, did not clearly show changes in canopy color in September and October (Figure 2(f)). This means that the RGwhtB method was less advantageous than the RGrgbyB method in terms of discriminating pixels representing rice plant, soil, and other objects. As shown in Table 1, the two methods visually demonstrated differences in clustering the pixels of the Microsoft Office gamut (Figures 2(f) and 2(h)).

**Figure 4:**
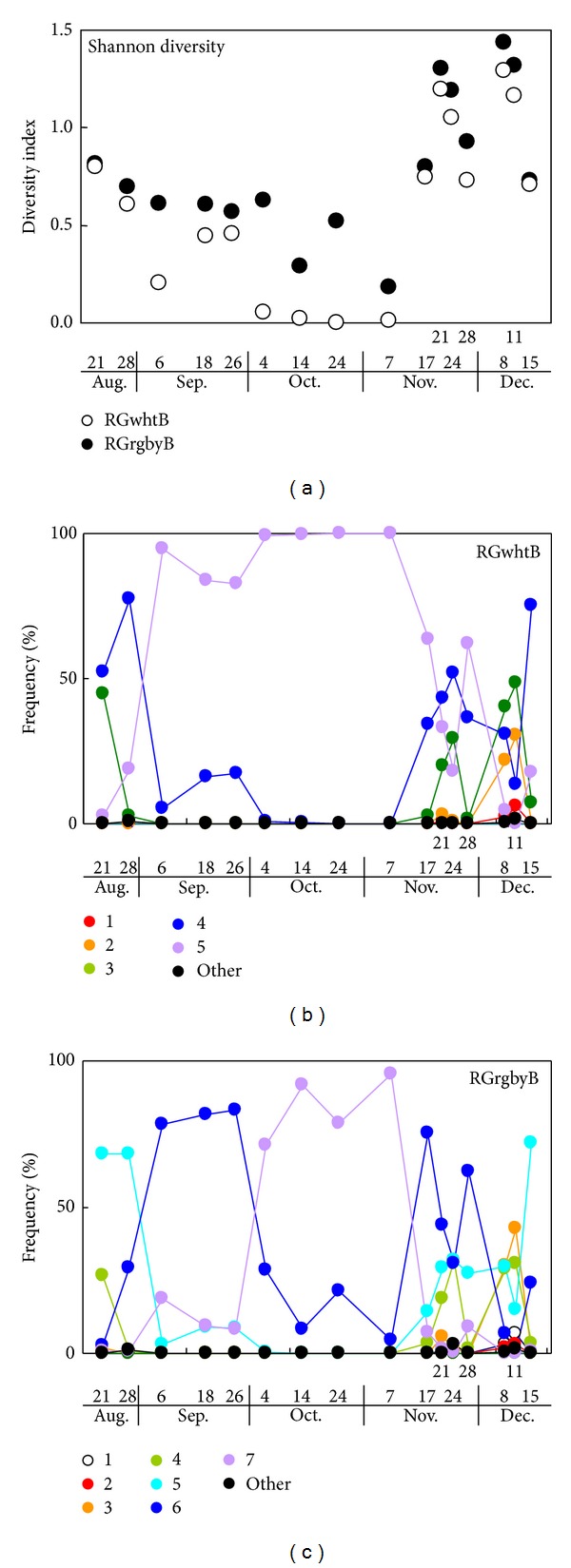
Shannon diversity of distribution patterns of pixel color clusters in the multi-temporally acquired rice canopy images (a) and appearance of pixel color clusters in the canopy image for the RGwhtB and the RGrgbyD methods ((b) and (c), resp.).

Figure 4 indicates the appearance and disappearance of the clusters in the target frame within the period of observation. For both methods, the cluster distribution patterns became complicated in late November when the rice grains were maturing and the leaves were experiencing senescence. When bare soil was observed in late August and mid-December the cluster distribution pattern for each method was simpler than in the maturity period. The color of the bare soil was largely described by color clusters 4 (RGwhtB method) and 5 (RGrgbyB method) with a large R intensity (>205) and a small G intensity (<48) (Table 1). In the RGrgbyB target frames for September (Figure 2(h)), relatively brighter pixels (cluster 6, R = 130, G = 114, Table 1) became dominant. In October, the dominance was taken over by darker pixels (cluster 7, R = 106, G = 103, Table 1). In the RGwhtB target frames, this change in dominant color was not shown (Figure 2(f)), whereas, in September and October, the most dominant was cluster 5 (R = 127, G = 113, Table 1). Eventually, changes in the color profile in the target frame within the two months (September and October) were apparently undetectable for the RGwhtB method, implying that the pixels representing different objects could not be discriminated. A marked difference between the methods was that the RGrgbyB method generated clusters 6 and 7 to indicate different time periods in September and October which were difficult to discriminate when employing the RGwhtB method.

These periods are thought to be the vegetative and reproductive stages according to the FAO's manual [26]. Patterns of cluster distribution in the target frames on 26 September and before in Figure 2(h) suggest the vegetative stage [18]. Likewise, the cluster distribution patterns on 4, 14, and 24 October and 7 November are thought to represent the midseason stage [18]. Therefore, the patterns for 17 November to 11 December are likely to be of the late season stage. As described later, the vegetative stage defined in the FAO's manual was further divided in this study. 

Values of Shannon diversity of color cluster distribution were determined for the dates. According to changes in the Shannon diversity index, the target frames in the RGrgbyB image had greater values of the Shannon diversity index within the period, especially in the midseason stage, than those derived from the RGwhtB image (Figure 4). Canopy diversity may be used as an information source on field crop production though the application was not found. Canopy diversity, however, was proven to be a good indicator for forest management. For example, changes in the value of the diversity index for the pine canopy significantly described pine biomass production [27]. Because spatiotemporal variability is an important issue in crop production, and the current approach is feasible to employ to observe areas of crop production, crop canopy diversity is worth being investigated to determine what the indices indicate. With the current method, balloons [28] or unmanned planes [29] may help in identifying the spatiotemporal color changes of plant organs.

**Figure 5:**
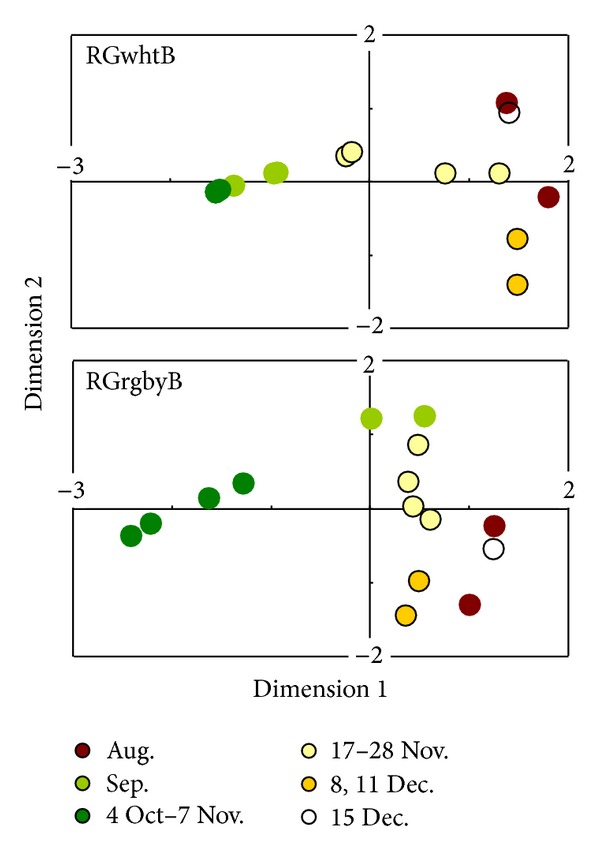
Multidimensional scaling plots to score distribution patterns of the pixel color clusters in the target rice canopy frame.

Figure 5 demonstrates the proximity among the cluster distribution patterns in the target frames in Figure 2(f) or Figure 2(h). The vegetative stage (before 4 October) can be divided into the first and second substages according to the cluster distribution patterns for the RGrgbyB method (Figure 2(h)). However, for the RGwhtB method, cluster distribution patterns in the second vegetative (September) and midseason (4 October to 7 November) stages were scored close to each other (Figure 5). The RGwhtB method was disadvantageous to describe the changes between these (sub-) stages (Figure 2(f)). As shown in this example, it is not always suitable to regard a single rice season as having three abruptly divided stages. Hence, the continuous observation of changes in canopy color enables flexible farming practices that respond to the changes in color [2].

**Figure 6:**
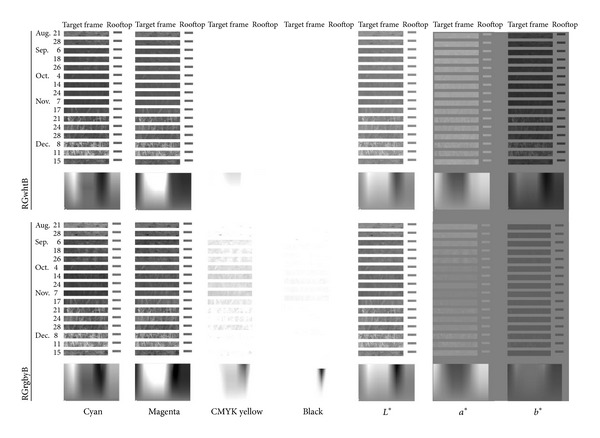
Grayscale images of the target rice canopy and the gamut derived from the pseudo-RGB images (Figures 2(f) and 2(h)).

To find the cause of the superiority of the RGrgbyB method, C, M, Y, K, L*, a*, and b* grayscale images were prepared from the RGrgbyB and RGwhtB JPEG images (Figure 6). The most marked difference was that the Y grayscale image derived from the RGrgbyB image showed lower intensity values, that is, darker pixels, in the target frames for 4 October to 7 November whereas those for the other time points had greater intensity values. This was thought to be the reason why the RGrgbyB method was more effective at finding the differences between the vegetative (September) and midseason (4 October to 7 November) stages than the RGwhtB method. In the RGB color model, yellow and blue have a complementary relationship [30]. Therefore, if a B grayscale image of an RGB digital photograph is replaced with a white mat, the intensity of B becomes 255 for all pixels. Then, the information on the intensity of CMYK yellow as the complementary color must be largely lost (Figure 6). At the same time, the relative intensity of B to that of yellow becomes larger. This results in decreases in values of b* for pixels in the b* grayscale image because b* is a color component that shows the relative intensity of Y to B (Figure 3, [25]). This made the entire b* grayscale image for the RGwhtB method darker than that for the RGrgbyB method (Figure 6).

## 4. Conclusions

The quantification of temporal color changes in a rice canopy was enabled by pixel clustering based on the current pseudo-RGB methods. The RGrgbyB method more precisely revealed the temporal color changes in the rice canopy that indicate rice growth stages than the RGwhtB method. The relative advantage of the RGrgbyB method was suggested by the greater sensitivity to show differences in the (intensity) values of color components, especially yellowness. The greater sensitivity was thought to be associated with the greater Shannon diversity values for the distribution patterns of pixel color clusters in the area of the target canopy and the greater power to discriminate the cluster distribution patterns for rice growth stages. The RGrgbyB method indicated the first and second substages in the vegetative stage. However, the RGwhtB method did not discriminate between the second vegetative and midseason stages. The current pseudo-RGB methods are worth being examined and applied in the observation of (spatio-)temporal changes in the colors of various plant species/organs in actual fields where brightness varies significantly with time.

## Figures and Tables

**Table 1:** Mean (intensity) values of the color components for major pixel color clusters.

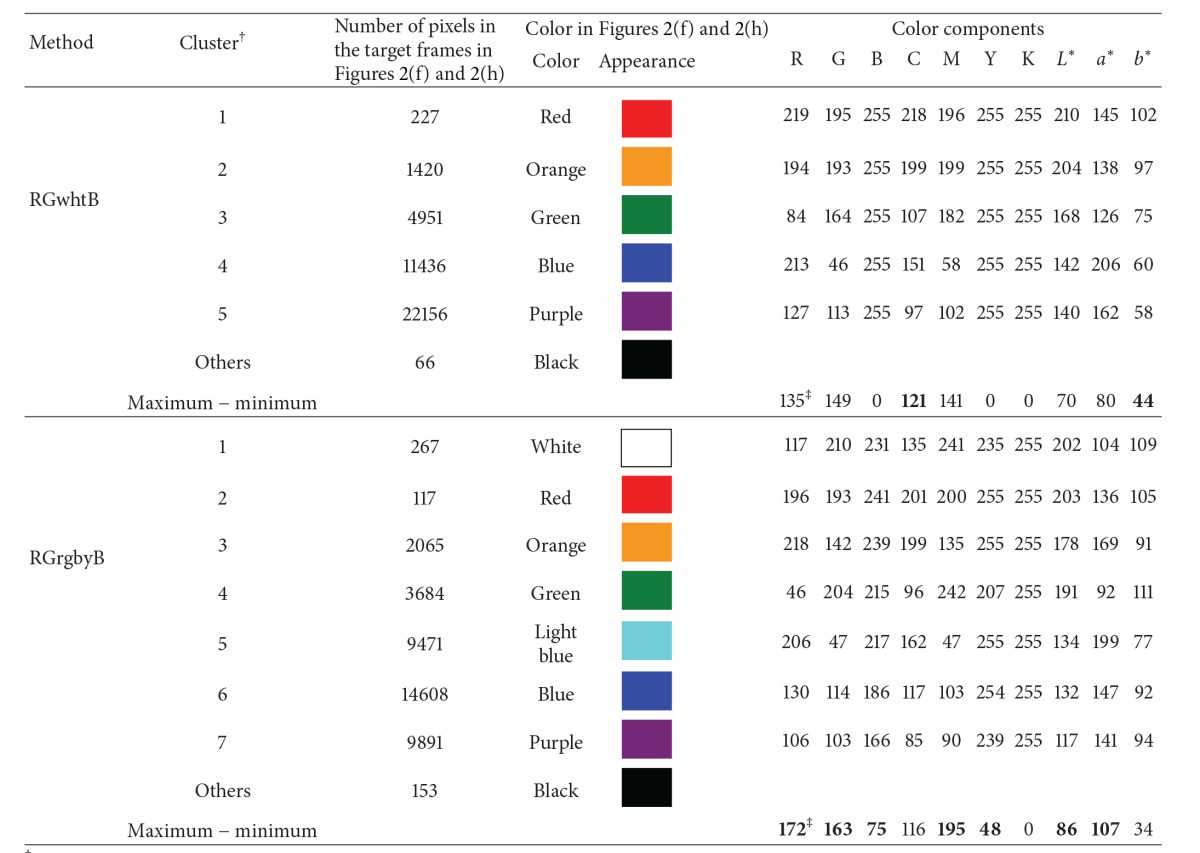

^†^Clusters with pixel numbers >100 in the 16 target frames for 21 August to 15 December 2011 (Figure 2).

^‡^For maximum − minimum (range), the bold values are larger for either method than the other.
